# Decade-long antiretroviral therapy in Uganda: Population-health outcomes from a national HIV treatment cohort, 2014–2024

**DOI:** 10.1371/journal.pgph.0005906

**Published:** 2026-03-16

**Authors:** Maria Magdalene Namaganda, Stathis Gennatas, Laura Merson, Esteban Garcia, Tom Edinburgh, Joyce Nakatumba Nabende, David Patrick Kateete, Charles Batte, Misaki Wanyengera, Daudi Jjingo, Florence Kivunike, Yunus Miya, Darius Kato, Simple Ouma, Susie Welty, Hussein Mukasa Kafeero, Gerald Mboowa

**Affiliations:** 1 Department of Immunology and Molecular Biology, School of Biomedical Sciences, College of Health Sciences, Makerere University, Kampala, Uganda; 2 Institute for Global Health Sciences, University of California, San Francisco, California, United States of America; 3 International Severe Acute Respiratory and Emerging Infectious Consortium, Pandemic Sciences Institute, University of Oxford, Oxford, United Kingdom; 4 Department of Computer Science, School of Computing and Information Technology, Makerere University, Kampala, Uganda; 5 Lung Institute, School of Medicine, College of Health Sciences, Makerere University, Kampala, Uganda; 6 African Center of Excellence in Bioinformatics and Data-Intensive Sciences, Makerere University, Kampala, Uganda; 7 The AIDS Support Organization (TASO), Kampala, Uganda; 8 Department of Medical Microbiology, Habib Medical School, Faculty of Health Sciences, Islamic University in Uganda, Kampala, Uganda; University of Cape Town Faculty of Health Sciences, SOUTH AFRICA

## Abstract

The scale-up of antiretroviral therapy (ART) has transformed the HIV epidemic in sub-Saharan Africa, yet longitudinal public health insights from national programmatic cohorts remain scarce. As global targets move toward epidemic control and elimination of virological failure, understanding how profiles and outcomes of people living with HIV (PLWH) evolve in real-world settings is essential. The AIDS Support Organisation (TASO), Uganda’s largest HIV care provider, maintains one of the longest-running national registries of PLWH. We aimed to generate insights to inform targeted interventions, optimize programmatic responses and improve long-term care for PLWH in resource-constrained settings. We conducted a secondary analysis of routinely collected data within an open cohort of 54,348 PLWH enrolled at 11 TASO clinics between 2014–2024. Descriptive statistics summarized demographic, clinical, immunological, behavioral and socioeconomic characteristics at baseline and follow-up. At most recent recorded follow-up, 60.4% of PLWH were female with median age of 39 years (IQR 31–47); 55% aged 25–44 years. At ART initiation, non-nucleoside reverse transcriptase inhibitor (NNRTI)-based regimens dominated (>90% through 2017), by 2024, > 99% of ART initiations were dolutegravir (DTG)- based, aligning with national policy. Immune recovery improved over study period; however, 12% still presented with CD4 < 200 cells/µL. Most PLWH initiated care in WHO stage I–II, and prevalence of advanced disease declined further at last record. Self-reported adherence exceeded 95%. Tuberculosis was the most frequent infectious comorbidity (5% prior history), whereas hypertension (3.9%) and diabetes (2.5%) were the leading non-communicable conditions. Socioeconomic vulnerability reported 57% with irregular income and 21% unemployed. Psychosocial stressors, including poverty and stigma, were common. Findings show progress alongside persistent gaps and structural barriers. They mandate earlier diagnosis, integrated tuberculosis and non-communicable disease management, strengthened socioeconomic support, and risk-stratified care using predictive modeling to reduce virological failure and drive Uganda toward 95–95–95 targets in resource-constrained settings.

## Introduction

The scale-up of antiretroviral therapy (ART) in sub-Saharan Africa (SSA) represents one of the greatest public health achievements of the past two decades [1]. Guided by the Joint United Nations Programme on HIV/AIDS (UNAIDS) 95–95–95 target now due [2], countries have expanded testing, treatment coverage, and viral-load monitoring, yielding large gains in survival and population-level viral suppression. Uganda, one of the first countries in Africa to recognize the HIV/AIDS epidemic in the early 1980s [3], has been at the forefront of this response. Since the earliest descriptions of “Slim disease” in 1984, through to the adoption of universal test-and-treat (UTT) in 2017 and the transition to dolutegravir (DTG)-based regimens from 2018, Uganda’s ART program has undergone major policy transitions that have reshaped the landscape of HIV care [4]. These policy milestones, alongside the introduction of routine viral load monitoring in 2014 [5], have transformed survival, viral suppression, and quality of life for people living with HIV (PLWH). Yet sustaining these gains requires an equally strong understanding of how profiles of PLWH have evolved over time, particularly in resource-limited settings where structural and behavioral barriers to care persist, and how they may shape long-term treatment outcomes such as HIV virological failure and drug resistance.

The AIDS Support Organization (TASO), initially formed in 1987 to offer access counselling and testing, has been central to Uganda’s HIV response. As the country’s largest provider of HIV care, TASO operates 11 centers nationwide and community outreach platforms that collectively serve tens of thousands of PLWH. TASO’s data registry is notably rich for the region, beyond demographics and ART data, it integrates immunologic and clinical staging, anthropometrics, adherence indicators, comorbidities and opportunistic infections, and socioeconomic and psychosocial variables. Importantly, the registry includes routine fields for selected non-communicable diseases (NCDs) and other comorbidities alongside opportunistic infections—information that is often sparse or inconsistently captured in many HIV care registries in the region. As PLWH age and multimorbidity becomes more common, these domains are increasingly relevant for programme planning and service integration. In this study, we describe the recorded availability and burden of these comorbidity fields as part of the cohort profile, while recognising the limitations of routine documentation.

This breadth allows not only tracking of biomedical effects of policy shifts, such as replacement of non-nucleoside reverse transcriptase inhibitor (NNRTI) regimens with Dolutegravir (DTG-based) regimens, but also characterization of structural and behavioral vulnerabilities that influence long-term outcomes in resource-limited settings.

Cohort descriptions from East Africa often examine a single domain such as regimen patterns or CD4 reconstitution or restrict analyses to short follow-up windows, leaving few integrated, longitudinal portraits to support predictive modeling of treatment outcomes, particularly virological failure [6]. In addition, controversies remain regarding the optimal management of PLWH who fail on ART, particularly in resource-constrained settings where treatment options may be limited [7,8]. Some studies advocate early regimen changes in response to virological failure (VF), while others emphasize intensive adherence support and retention before switching therapies. These divergences highlight the need for context-specific evidence to inform clinical decision-making and improve PLWH outcomes. Compounding this, routine information systems [9] are optimized for retrospective reporting rather than real-time performance management or risk prediction, limiting their utility for anticipatory interventions.

Against this backdrop, we analyzed records of PLWH receiving care at TASO-Uganda clinics from 2014–2024 to characterize their demographic, clinical, and treatment profiles. We described temporal trends in ART initiation and regimen composition; immunological and WHO clinical staging; body mass index; treatment adherence; comorbidities; and socioeconomic characteristics. This multi-domain portrait of Uganda’s ART program over a decade provides actionable evidence for national programming and regional strategy, and it establishes a foundation for subsequent predictive modeling of outcomes particularly virological failure.

## Materials and methods

### Ethics statement

Ethical approval was obtained from the Research Ethics Committee, School of Biomedical Sciences (SBS-REC), Makerere University, under study number SBS-2024–539. The SBS-REC Committee waived the requirement for informed consent because the study involved a retrospective review of de-identified electronic health records. No direct contact with PLWH or collection of additional data was undertaken, and the study posed no risk to privacy or confidentiality.

Additional approval was obtained from the Uganda National Council for Science and Technology (UNCST), reference number HS3982ES. The AIDS Support Organization (TASO) granted administrative clearance (reference TASO REC/ADMC11/2024-UG-REC-009). All data were de-identified before analysis; direct identifiers were removed and replaced with study IDs. The re-identification key is securely held by TASO and was not accessible to the analysts. Data abstraction for this analysis was performed on 10 December 2024.

### Study design and cohort type

We conducted a secondary analysis of routinely collected programme data from The AIDS Support Organisation (TASO) in Uganda. The dataset forms an open, longitudinal cohort: individuals entered at enrolment or ART initiation and contributed variable follow-up data until their last recorded clinic visits or laboratory result within the observation window (1 January 2014 – 31 October 2024). This window was pre-specified to align with the period of routine viral load monitoring scale-up and to support a decade-long cohort description. The initial client-level data from the TASO Electronic Medical Records (EMR) contained 54,415 clients; 67 clients with recorded ART initiation dates prior to 1 January 2014 were excluded, yielding a final analytic cohort of 54,348 individuals. Data abstraction for this analysis was performed on 10 December 2024. For descriptive summaries, we defined “baseline” as the earliest available record for a given variable within the observation window, and “most recent” follow-up as the latest available record within the window. This 10-year period is programmatically significant, spanning the World Health Organization’s 2014 call for routine viral load monitoring and Uganda’s successive ART policy milestones, and thus provides a robust decade-long perspective on HIV care trajectories.

### Setting and data source

TASO is Uganda’s largest HIV service provider, operating 11 Centres of Excellence that serve both urban and rural catchments across Uganda: TASO Entebbe, TASO Gulu, TASO Jinja, TASO Masaka, TASO Masindi, TASO Mbale, TASO Mbarara, TASO Mulago, TASO Rukungiri, TASO Soroti, and TASO Tororo. TASO delivers comprehensive HIV care within Uganda’s national HIV programme framework, including ART initiation and follow-up, routine clinical assessments, and laboratory monitoring (including viral load testing in line with national guidance). Clinical and programme information is captured during routine care in site-level electronic medical record (EMR) systems, which feed a harmonised registry. The registry includes demographics, ART and regimen history, WHO clinical staging, selected laboratory results (e.g., CD4 and viral load where available), anthropometrics, adherence measures, opportunistic infections, selected comorbidities, and socioeconomic and psychosocial fields; completeness of some domains may vary by site and time as part of routine service delivery. TASO’s HIV care and support services are predominantly donor-supported and are generally provided at no cost to clients at the point of care, although availability and resourcing for some ancillary services may vary by site and over time [10].

### Study population and eligibility

All PLWH registered for care at any TASO site between 2014–2024 were eligible and we applied a census approach (no sampling). Eligibility required enrolment at a TASO site within the observation window. For domain specific analyses, we used available-case approach: analyses requiring viral load (VL) were restricted to individuals with ≥1 VL result in the registry, and analyses of initiation measures (e.g., baseline ART class or baseline CD4), excluded records missing the relevant baseline field for that analysis only. After cleaning, 54,348 unique PLWH were available for descriptive summaries.

### Variables and operational definitions

We summarised five domains (full variable list in Table 1/ S1 Data):

**Table 1:** Demographic characteristics.

Variable	N	N = 54,348^1^
**ART_Start_Year**	54,348	
2014		9,000 (17%)
2015		5,164 (9.5%)
2016		4,082 (7.5%)
2017		5,711 (11%)
2018		10,577 (19%)
2019		5,537 (10%)
2020		4,458 (8.2%)
2021		2,874 (5.3%)
2022		2,602 (4.8%)
2023		2,707 (5.0%)
2024		1,636 (3.0%)
**Health_Site**	54,348	
Entebbe		4,829 (8.9%)
Gulu		951 (1.7%)
Jinja		5,568 (10%)
Masaka		7,114 (13%)
Masindi		3,639 (6.7%)
Mbale		6,288 (12%)
Mbarara		5,832 (11%)
Mulago		6,989 (13%)
Rukungiri		4,784 (8.8%)
Soroti		2,491 (4.6%)
Tororo		5,863 (11%)
**Age(most_recent)**	54,348	39.9 [32.3, 48.9]
**RecentAge_Group**	54,348	
0-1		3 (<0.1%)
1-14		1,442 (2.7%)
15-19		1,027 (1.9%)
20-24		1,771 (3.3%)
25-44		29,733 (55%)
45-60		16,482 (30%)
60+		3,890 (7.2%)
**Initiation_Age**	54,335	33.3 [26.2, 41.8]
(NA)		13
**Initiation_Age_Group**	54,335	
0-1		296 (0.5%)
1-14		2,751 (5.1%)
15-19		1,387 (2.6%)
20-24		5,493 (10%)
25-44		33,422 (62%)
45-60		9,585 (18%)
60+		1,401 (2.6%)
(NA)		13
**Gender**	54,348	
F		32,843 (60%)
M		21,505 (40%)
**Religion**	52,653	
Anglican		17,819 (34%)
Catholic		22,441 (43%)
Muslim		6,665 (13%)
Orthodox		60 (0.1%)
Unknown		827 (1.6%)
Pentecostal		3,900 (7.4%)
Protestant		333 (0.6%)
SDA		608 (1.2%)
(NA)		1,695
^1^n (%); Median [Q1, Q3]		

**Note**: Age at ART initiation was calculated from date of birth and the recorded ART start date. (NA) indicates records where the ART start timing met the inclusion criterion (ART start during 2014–2024) but the ART start value was not recorded as a complete/parseable date (e.g., year-only or incomplete date), so age at initiation could not be computed. Age at recent record was calculated from date of birth and the data-freeze date (10 December 2024).

1. Demographics: sex, age at recent record (years), age groups (0–1, 1–14, 15–19, 20–24, 25–44, 45–60, ≥ 60), health site.
2. Clinical status included CD4 (cells/µL) at baseline and most recent; categories <200, 200–500, > 500, WHO clinical stage (I–IV) at baseline and most recent follow-up and BMI (kg/m²) derived from weight/height and categorized as <18.5, 18.5–24.9, 25.0–29.9, ≥ 30.0. BMI was only computable where both height and weight were recorded; therefore, BMI summaries reflect clients with documented measurements and should be interpreted in light of missingness. Nutrition status was extracted as the routine triage category recorded at clinic visits based on mid–upper arm circumference (MUAC) screening and documented as colour codes: green (normal), yellow/orange (moderate acute malnutrition risk), and red (severe acute malnutrition). Adherence data was extracted as recorded in the TASO EMR at follow-up visits (e.g., good/fair/poor; Table 2). This categorisation is primarily based on client self-report, where a health worker asks the client whether they took their ART as prescribed without missing doses since the last appointment. Where pill counts are performed, adherence was estimated from the proportion of dispensed pills taken. We report denominators because adherence was not recorded for all clients or visits.
3. Treatment variables included baseline and current ART class derived from regimen anchors (INSTI-based, NNRTI-based, protease inhibitor (PI)-based); ART start year and duration on ART (years from ART start to last contact within window). Policy eras were used for narrative contextual interpretation: 2014–2016 (VL scale-up), 2017–2019 (UTT adoption), 2020–2024 (DTG consolidation).
4. Comorbidities and opportunistic infections (OI): tuberculosis (TB) status (on treatment, diagnosed, presumptive, completed, none/unknown), history of TB treatment, diabetes, hypertension, cancer (and type), and EMR “Underlying OI” categories. Comorbidities and opportunistic infections were summarised as recorded in the EMR within the observation window; incident versus prevalent timing could not be consistently distinguished due to incomplete diagnosis dates in the extract.
5. Behavioural/psychosocial and socio-economic: nutrition triage (green/yellow/red), alcohol and smoking status, receipt of psychosocial support, depression screening (yes/no/unknown), psychosocial problem categories (PSS), education (initial, recent), employment (detailed) and collapsed employment status (Regular income/ Irregular income/ Unemployed), marital status (detailed and collapsed married vs unmarried). For time-varying characteristics such as marital status and employment, we report values at the most recent recorded entries within the study.
6. Virological outcome measures and definitions: Viral load (VL) results were summarised in line with consolidated guidelines for the prevention and treatment of HIV in Uganda. Initial VL was defined as the first viral load measurement after a minimum of 6 months on ART. Viral suppression was defined as VL < 1,000 copies/mL and non-suppression as VL ≥ 1,000 copies/mL; virological failure was defined as two consecutive VL measurements ≥1,000 copies/mL following intensive adherence counselling (IAC). [11]. VL data extracted for this analysis from the TASO EMR were available as Initial_VL and up to three subsequent VL measurements (VL_3, VL_2, VL_1), alongside the latest recorded VL (Recent_VL) within the study period. For programme outcome summaries, we report viral suppression (VL < 1,000 copies/mL) versus non-suppression (VL ≥ 1,000 copies/mL); these correspond to the fields labelled ‘success’ and ‘failure’ in the dataset. For further descriptive profiling, VL was categorised as ≤50 copies/mL (undetectable), and low-level viraemia was reported in two ranges (51–199 and 200–999 copies/mL) to distinguish lower- and higher-range detectable viraemia below the programme non-suppression threshold. Denominators are reported for all VL summaries because VL testing was not recorded for all clients; repeat-measure patterns are described where available.

**Table 2:** Clinical characteristics.

Variable	N	N = 54,348^1^
**Baseline_BMI**	11,562	21.8 [19.0, 25.4]
(NA)		42,786
**Baseline_BMI_Category**	11,562	
Underweight		2,503 (22%)
Normal		5,976 (52%)
Overweight		1,776 (15%)
Obese		1,307 (11%)
(NA)		42,786
**Recent_BMI**	30,435	23.0 [20.1, 27.0]
(NA)		23,913
**Recent_BMI_Category**	30,435	
Underweight		4,127 (14%)
Normal		15,479 (51%)
Overweight		6,482 (21%)
Obese		4,347 (14%)
(NA)		23,913
**Baseline_WHO_Stage**	49,590	
WHO_1		14,385 (29%)
WHO_2		29,384 (59%)
WHO_3		2,439 (4.9%)
WHO_4		720 (1.5%)
Unknown		2,662 (5.4%)
(NA)		4,758
**Current_WHO_Stage**	53,052	
Unknown		338 (0.6%)
WHO_1		14,692 (28%)
WHO_2		35,800 (67%)
WHO_3		1,607 (3.0%)
WHO_4		615 (1.2%)
(NA)		1,296
**Adherence**	47,468	
Fair		589 (1.2%)
Good		45,939 (97%)
Poor		710 (1.5%)
Unknown		230 (0.5%)
(NA)		6,880
**Nutrition**	51,505	
Unknown		1,814 (3.5%)
Green		47,801 (93%)
Red		416 (0.8%)
Yellow		1,474 (2.9%)
(NA)		2,843
**Baseline_CD4**	20,305	385.0 [205.0, 589.0]
(NA)		34,043
**Baseline_CD4_Category**	20,305	
<200		4,826 (24%)
200-500		9,028 (44%)
>500		6,451 (32%)
(NA)		34,043
**Recent_CD4**	18,494	473.0 [274.0, 703.0]
(NA)		35,854
**Recent_CD4_Category**	18,494	
<200		3,388 (18%)
200-500		6,559 (35%)
>500		8,547 (46%)
(NA)		35,854
^1^Median [Q1, Q3]; n (%)		

**Note:** Nutrition status (MUAC triage): green = normal nutritional status; yellow = moderate acute malnutrition risk; red = severe acute malnutrition.

### Data processing and quality control

Data were extracted from site EMRs as spreadsheets and merged after structural harmonisation. Cleaning included: type standardisation (dates parsed as Date; numerics coerced; categorical values trimmed/case-standardised with harmonized labels); de-duplication (repeated rows on the same person and date collapsed; ID conflicts resolved using a composite of site ID, sex, year of birth, and first visit date); and range/logic checks (physiologically implausible values were set to missing, examples: height <120 or >220 cm; weight <20 or >200 kg; BMI < 10 or >60; CD4 < 0 or >2,000; dates in the future or pre-1980; negative VL). A data-freeze on 10 December 2024 locked the analysis dataset; where multiple records occurred on the same date, we retained the most complete record for that variable. All data transformations were implemented using scripted, version-controlled code for reproducibility.

### Statistical analysis

This study employed a descriptive analytical design. Continuous variables were summarised using medians and interquartile ranges (IQRs), and where helpful their distributions were illustrated with violin and box-plot overlays. Categorical variables were described as counts and percentages overall and, when relevant, stratified by years of ART initiation. For comparisons across time, we summarised participant variables at two descriptive anchor points; baseline (earliest record within the observation window) and most recent follow-up (latest record within the window).

Data completeness varied by variable and over time in the routine TASO EMR extract; we therefore used available-case analysis for descriptive summaries, reporting variable-specific denominators and missingness, and no imputation was performed.

Visualisations included stacked proportional bar charts to display annual distributions of ART class, CD4 categories, WHO clinical stages, body-mass-index categories, and adherence levels. For ease of comparison, faceted panels distinguished “at initiation” from “most recent follow-up” while maintaining identical colour schemes and category ordering across panels.

**Fig 1:**
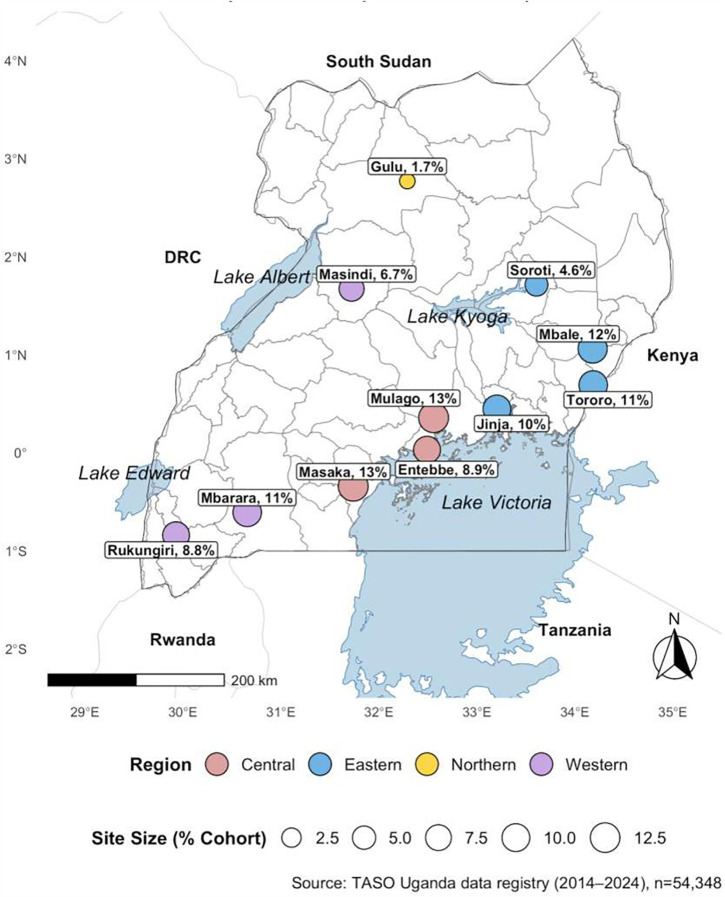
Regional distribution of TASO Uganda sites and proportion of PLWH receiving care (2014–2024 open cohort data). Map showing the geographic distribution of the 11 TASO centres of excellence across Uganda and the proportion of cohort participants served by each site (n = 54,348). Administrative boundaries and water bodies were sourced from Natural Earth (Admin 0 – Countries; Admin 1 – States/Provinces; and Lakes). Natural Earth data are in the public domain and compatible with the CC BY 4.0 license. The map was rendered in R using the sf package. Base layer source (Natural Earth): https://www.naturalearthdata.com/downloads/ License/terms (Public domain): https://www.naturalearthdata.com/about/terms-of-use/.

All analyses were conducted in R version 4.5.0, using the readxl, dplyr, tidyr, forcats, ggplot2, gtsummary, and companion packages. Spatial rendering of the national service footprint (Fig 1) employed the sf package. All analytic scripts were version-controlled, and a de-identified version is available from the authors upon reasonable request for peer review.

To contextualize temporal trends, we stratified analyses by programmatic periods aligned with national guideline milestones and policy eras: the pre–Treat All era, also VL roll out era (2014–2016), the Universal Test and Treat (UTT) scale-up (2017–2019), and the dolutegravir (DTG) consolidation phase (2020–2024).

This manuscript is descriptive; inferential and predictive modelling analyses are outside the scope of this report.

## Results

### Service delivery and geographic reach

TASO’s national service network spans all four major regions of Uganda: Central, Eastern, Northern, and Western, providing broad geographic access to HIV care. Among the 54,348 PLWH across the 11 TASO centres in this analysis (Table 1), the largest caseloads were recorded at TASO Masaka (13.1%), Mulago (12.9%), Mbale (11.6%), and Jinja (10.2%), followed by Mbarara (10.7%), Tororo (10.8%), Entebbe (8.9%), and Rukungiri (8.8%). Lower-volume sites included Masindi (6.7%), Soroti (4.6%), and Gulu (1.7%). These distributions reflect both Uganda’s historical HIV burden and the urban and peri-urban catchment of TASO’s high-volume centres, as presented in Fig 1.

Building on the above geographic context, the following sections describe the demographic, clinical, and treatment characteristics of the cohort.

### Demographic characteristics

Data from a total of 54,348 PLWH was analyzed across all TASO sites between 2014 and 2024. The cohort participants were predominantly female (60.4%, Table 1), median age was 39.9 years (IQR: 32.3–48.9), and the majority, 55% of individuals, were within the 25–44 age group, at the most recent recorded follow-up.

**Fig 2:**
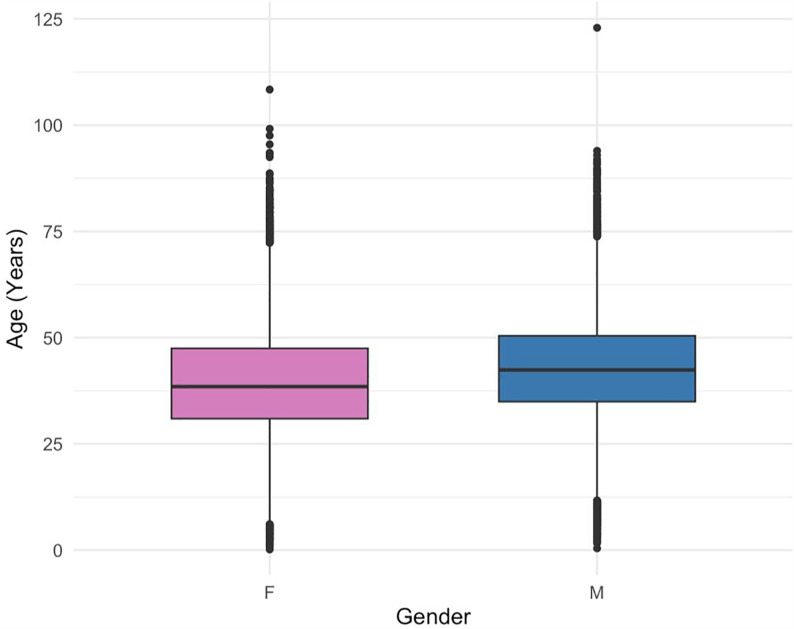
Age distribution by gender. Distribution of age by gender among individuals enrolled in care across TASO Uganda sites (2014–2024).

Boxplot analysis revealed a slightly older age profile among males compared to females (Fig 2).

**Fig 3:**
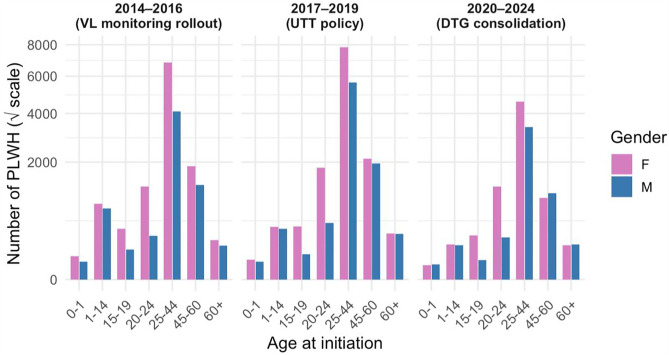
Shifts in ART initiation across HIV policy evolution periods in Uganda by age and gender. Across all three policy periods, women consistently initiated ART in greater numbers than men, particularly in the 20–44-year reproductive age range.

Age distribution at ART initiation by gender and policy band (2014–2016, 2017–2019, 2020–2024) showed that across all time periods, women consistently outnumbered men, particularly in the reproductive age brackets (Fig 3). Children and adolescents under 20 years represented a small but stable proportion of ART initiates over time.

### Clinical variables

#### WHO clinical stage.

The majority of PLWH entered care in early disease stages, 29% were WHO stage I and 59% stage II, while only a small proportion had advanced disease (5% stage 3 and 1.5% stage 4). A further 5.4% had unknown staging, and staging data were missing for 9% of PLWH.

By the most recent follow-up, 67% were classified as WHO stage 2 and 28% as stage 1, with advanced disease declining further to 3.0% (stage 3) and 1.2% (stage 4). Missing staging had decreased substantially to 2% (Table 2). This trend highlights that while the vast majority of PLWH were initiated on ART at early disease stages, ongoing clinical monitoring shows further consolidation of favorable staging profiles during follow-up, with advanced disease becoming rare (<5%).

#### Body mass index.

Among participants with BMI data (n = 11,562 at baseline; n = 30,435 at follow-up), the median BMI rose from 21.8 kg/m² (IQR 19.0–25.4) at ART initiation to 23.0 kg/m² (IQR 20.1–27.0) at the most recent visit (Table 2). At baseline, 22% were underweight, 52% had normal BMI, 15% were overweight, and 11% were obese. By follow-up, underweight prevalence declined to 14%, whereas overweight and obesity increased to 21% and 14%, respectively.

#### Nutritional status.

Programmatic nutrition screening mirrored the BMI profile: among 51,505 participants with a nutrition assessment, 93.0% were classified as green (nutritionally adequate), 2.9% as yellow (moderate risk), and 0.8% as red (poor nutrition) (Table 2).

#### Adherence.

Self-reported adherence at the most recent visit was high (Table 2): among 47,468 PLWH with available records, 97.0% reported good adherence, 1.2% fair, 1.5% poor, and 0.5% were recorded as unknown.

#### Immunological status (CD4).

**Fig 4:**
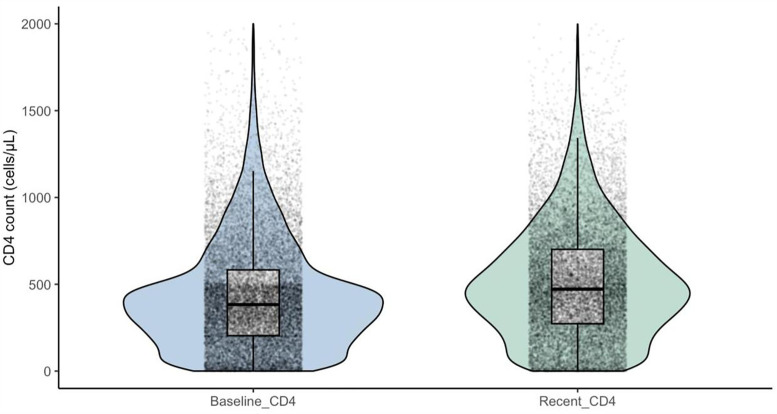
Shifts in CD4 count distributions from baseline to recent follow-up among PLWH in TASO Uganda cohort (2014-2024). Immunological status of people living with HIV (PLWH) at ART initiation and most recent follow-up, by WHO CD4 strata.

**Fig 5:**
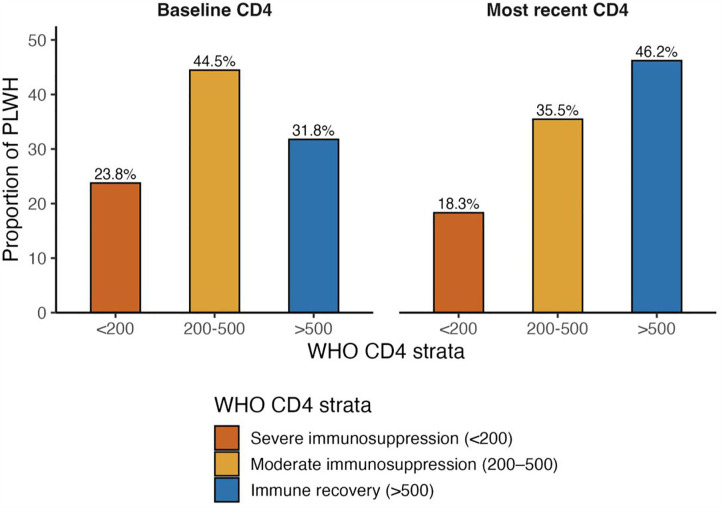
Immunological status of PLWH at ART initiation and most recent follow up, by WHO CD4 strata. Distribution of PLWH across WHO-defined CD4 categories at baseline (left panel) and most recent follow-up (right panel) in the TASO Uganda cohort, 2014–2024.

At ART initiation, the median CD4 cell count was 385 cells/µL (IQR 205–589), with 24% of PLWH starting treatment at <200 cells/µL (severe immunosuppression), 44% at 200–500 cells/µL (moderate immunosuppression), and 32% above 500 cells/µL (Table 2). At the most recent follow-up, the median CD4 count had risen to 473 cells/µL (IQR 274–703). The proportion of PLWH with CD4 > 500 cells/µL nearly doubled to 46%, while those with severe immunosuppression declined to 18%. (Figs 4 and 5).

### Treatment variables

#### Annual ART initiation trends.

**Fig 6:**
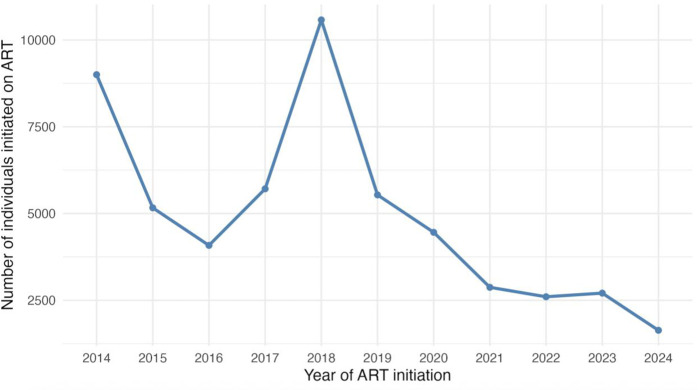
Trends in annual ART initiations among PLWH at TASO Uganda (2014-2024). Annual ART initiations among PLWH across all 11 sites at TASO, 2014-2024.

Annual ART initiations across all TASO sites displayed two distinct peaks over the decade. Approximately 9,000 individuals (17%) initiated therapy in 2014, coinciding with Uganda’s national rollout of routine viral-load monitoring that encouraged early ART uptake. After a dip during 2015–2016, initiations surged to 10,577 (19%) in 2018, reflecting nationwide adoption of the Universal Test and Treat strategy and rapid scale-up of dolutegravir-based regimens. From 2019 onward, new enrolments declined progressively to 1,636 (3%) by 2024, consistent with a maturing, largely saturated cohort and a programmatic shift toward sustaining long-term retention and viral suppression, Table 3 and (Fig 6).

**Table 3:** Treatment characteristics.

Variable	N	N = 54,348^1^
**ART_Start_Year**	54,348	
2014		9,000 (17%)
2015		5,164 (9.5%)
2016		4,082 (7.5%)
2017		5,711 (11%)
2018		10,577 (19%)
2019		5,537 (10%)
2020		4,458 (8.2%)
2021		2,874 (5.3%)
2022		2,602 (4.8%)
2023		2,707 (5.0%)
2024		1,636 (3.0%)
**Line_Current_ART**	54,342	
Unknown		18 (<0.1%)
1st_Line		52,753 (97%)
2nd_Line		1,534 (2.8%)
3rd_Line		37 (<0.1%)
(NA)		6
**Baseline_ART_Class**	53,226	
INSTI-based		15,831 (30%)
NNRTI-based		34,533 (65%)
PI-based		568 (1.1%)
Unknown		2,294 (4.3%)
(NA)		1,122
**Current_ART_Class**	54,235	
INSTI-based		41,090 (76%)
NNRTI-based		11,991 (22%)
PI-based		819 (1.5%)
Unknown		335 (0.6%)
(NA)		113
^1^n (%)		

### Line of regimen

By the most recent follow-up, majority, 52,753 (97%) were on first-line ART, 1,534 (2.8%) had switched to second-line, and only 37 (<0.1%) were receiving third-line therapy.

### Antiretroviral regimen evolution

At the cohort level (Table 3), 65% of PLWH initiated ART on non-nucleoside reverse transcriptase inhibitor (NNRTI)–based regimens, 30% on integrase strand transfer inhibitor (INSTI)–based regimens, and 1.1% on protease inhibitor (PI)–based regimens. By their most recent clinic visit, this pattern had reversed: 76% were on INSTI-based regimens, 22% on NNRTI-based regimens, and 1.5% on PI-based regimens, which proportions include long-treated clients alongside a near-universal shift to DTG–containing therapy over the decade.

**Fig 7:**
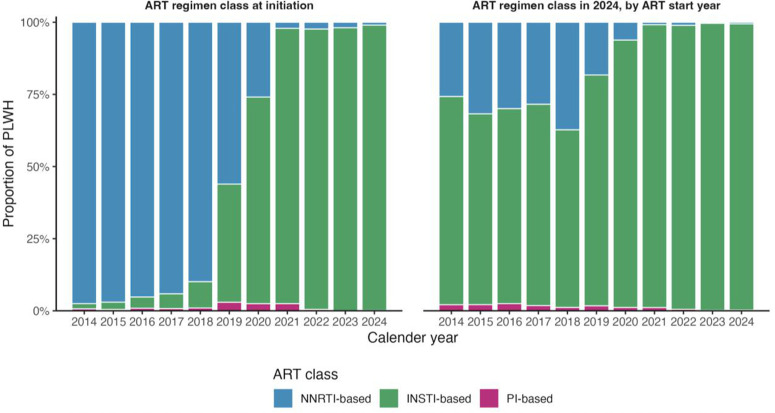
Evolution of ART regimen classes by study years, 2014-2024.

Fig 7 illustrates how this transition unfolded. The left panel shows ART regimen class at initiation by calendar year: NNRTI-based regimens predominated 2014–2018, then declined sharply following Uganda’s adoption of DTG in 2018. By 2020 onward, nearly all new initiations were INSTI-based. The right panel depicts the most recent ART regimen class in 2024, stratified by year of ART start. Irrespective of start year, the majority of PLWH had transitioned to INSTI-based regimens by 2024, reflecting comprehensive national rollout and policy consolidation. PI-based regimens remained rare across both initiation and current treatment distributions.

### Virological outcomes

Viral load results were available for a subset of the cohort during follow-up. Overall, 27,599 clients had an Initial_VL recorded and 33,384 had a Recent_VL recorded within the study period; VL was not recorded for 26,749 and 20,964 clients, respectively. At Initial_VL, 21,750/27,599 (78.8%) had undetectable viral load (≤50 copies/mL), 2,368 (8.6%) had VL 51–199 copies/mL, 1,120 (4.1%) had VL 200–999 copies/mL, and 2,361 (8.6%) were non-suppressed (≥1,000 copies/mL). At Recent_VL, 27,170/33,384 (81.4%) were undetectable, 2,744 (8.2%) had VL 51–199 copies/mL, 1,325 (4.0%) had VL 200–999 copies/mL, and 2,145 (6.4%) were non-suppressed.

Using the programme threshold of 1,000 copies/mL, viral suppression (VL < 1,000 copies/mL) was observed in 25,238/27,599 (91.4%) at Initial_VL and 31,239/33,384 (93.6%) at Recent_VL, while non-suppression (VL ≥ 1,000 copies/mL) was 2,361/27,599 (8.6%) and 2,145/33,384 (6.4%), respectively (Table 4).

**Table 4:** Virological outcomes.

VL category (copies/mL)	Initial VL, n (%) (N = 27,599)	Most recent VL, n (%) (N = 33,384)
≤50	21,750 (78.8)	27,170 (81.4)
51–199	2,368 (8.6)	2,744 (8.2)
200-999	1,120 (4.1)	1,325 (4.0)
≥1,000	2,361 (8.6)	2,145 (6.4)
**Treatment outcome (threshold 1000 copies/mL)**		
Suppressed (<1000)	25,238(91.4)	31,239(93.6)
Non-suppressed (≥1000)	2,361(8.6)	2,145(6.4)
VL not recorded, n	26,749	20,964

Notes: Percentages are calculated among clients with recorded VL results (N shown). ≤ 50 = undetectable; 51–199 and 200–999 = low-level viraemia; ≥ 1,000 = non-suppression. Initial and most recent VL summaries are based on different subsets and are not paired comparisons.

Because VL testing and documentation were not recorded for all clients, virological outcomes are reported in the context of monitoring completeness.

### Comorbidities and opportunistic infections

Comorbidity recording was heterogeneous but shows a dual burden of infectious and non-communicable disease in this long-term ART cohort (Table 5). Tuberculosis (TB) remained the leading opportunistic infection: 1.0% of participants were on TB treatment at their most recent visit, 0.3% had a current diagnosis without treatment, and 4.8% reported a prior TB episode. Other opportunistic infections were rare. Among 54,348 participants, respiratory infections were documented in 4.4%, dermatological conditions in 0.6%, digestive in 0.8%, neurological in 0.4%, and febrile illnesses in 0.4%. Cardiovascular, hematological, and oncological cases each accounted for fewer than 0.1%. Notably, 77% of entries in the “underlying opportunistic infection” field were classified as “other”, reflecting limitations in routine clinical coding.

**Table 5:** Comorbidities and opportunistic infections.

Variable	N	N = 54,348^1^
**Underlying_OI**	54,348	
Allergy/Immune		10 (<0.1%)
Cardiovascular		1 (<0.1%)
Dermatological		324 (0.6%)
Digestive		435 (0.8%)
Febrile Illness		206 (0.4%)
Gastrointestinal		51 (<0.1%)
Hematological		1 (<0.1%)
Mental Health		14 (<0.1%)
Musculoskeletal		167 (0.3%)
Neurological		223 (0.4%)
None/Unknown		8,163 (15%)
Oncology		8 (<0.1%)
Oral/ENT		124 (0.2%)
Other		41,846 (77%)
Reproductive/STI		282 (0.5%)
Respiratory		2,408 (4.4%)
Tuberculosis		85 (0.2%)
**TB_Status**	54,101	
Diagnosed_no_Treatment		6 (<0.1%)
On_TB_Treatment		542 (1.0%)
Diagnosed_TB		174 (0.3%)
No_TB		52,362 (97%)
Presumptive_TB		745 (1.4%)
Completed_Treatment		199 (0.4%)
Unknown		73 (0.1%)
(NA)		247
**TB_History**	47,568	2,276 (4.8%)
(NA)		6,780
**Diabetes**	50,525	1,274 (2.5%)
(NA)		3,823
**Hypertension**	51,268	2,003 (3.9%)
(NA)		3,080
**Cancer**	38,085	76 (0.2%)
(NA)		16,263
**Cancer_Type**	6,361	
Cervical_Cancer		52 (0.8%)
Laryngeal_Cancer		4 (<0.1%)
Oesophageal_Cancer		1 (<0.1%)
Leukemia		1 (<0.1%)
Unknown		26 (0.4%)
No_Cancer		6,277 (99%)
(NA)		47,987
^1^n (%)		

Notes: Conditions reflect diagnoses recorded at any time within the observation window, not necessarily baseline, unless otherwise stated. “Diagnosed_no_Treatment” reflects the programme status recorded in the EMR at the time of data capture and may include clients with a documented diagnosis whose corresponding treatment was not recorded in the same extract. “Other” reflects heterogeneous or non-standardized diagnostic entries in the TASO records.

Non-communicable diseases (NCDs) were present at lower but non-trivial levels, highlighting the epidemiologic transition accompanying long-term ART. Hypertension was self-reported in 3.9% and diabetes mellitus in 2.5% of participants, while cancers were rare (0.2%), with cervical cancer being the predominant type.

### Behavioral and psychosocial factors

Behavioural and psychosocial characteristics were variably documented across the cohort (Table 6).

**Table 6:** Behavioral and psychosocial characteristics.

Variable	N	N = 54,348^1^
**Alcohol_Status**	32,379	
Yes		1,320 (4.1%)
No		25,652 (79%)
Unknown		5,407 (17%)
(NA)		21,969
**Smoking_Status**	28,634	
No		22,852 (80%)
Unknown		5,568 (19%)
Yes		214 (0.7%)
(NA)		25,714
**Psychosocial_Support**	49,206	45,715 (93%)
(NA)		5,142
**Depression**	41,234	
Yes		3,593 (8.7%)
No		35,580 (86%)
Unknown		2,061 (5.0%)
(NA)		13,114
**PSS_Category**	54,348	
Alcohol And Substance Abuse		1,454 (2.7%)
Anxiety		608 (1.1%)
ART Readiness		1,407 (2.6%)
Bereavement		77 (0.1%)
Denial		1,062 (2.0%)
Dysfunctional Family		835 (1.5%)
Food Insecurity		37 (<0.1%)
Life Skills Deficiency		533 (1.0%)
Malnourished		38 (<0.1%)
No Issues Reported		4,808 (8.8%)
Non Disclosure		1,190 (2.2%)
Other		20,355 (37%)
Poverty		18,470 (34%)
Risky Sexual Behavior		1,151 (2.1%)
Spiritual Issues		109 (0.2%)
Stigma		988 (1.8%)
Stress		810 (1.5%)
Transition Challenges		416 (0.8%)
^1^n (%)		

Note: PSS categories are recorded per client per time point at data collection, reported here as mutually exclusive; concurrent psychosocial stressors may therefore be under-captured.

Among participants with recorded data on alcohol use (n = 32,379), 4.1% reported current alcohol consumption, 79% reported no use, and 17% were of unknown status. Cigarette smoking was infrequent: of 28,634 individuals with available information, only 0.7% reported smoking, while 80% reported no tobacco use and 19% had unknown status.

Psychosocial support services were widely accessed. Among 49,206 participants with documentation, 93% had received at least one form of structured psychosocial support. Depression screening was more limited (n = 41,234), but 8.7% had a recorded diagnosis of depression and 5% were classified as unknown.

Programmatic psychosocial assessments (PSS categories) highlighted structural and life-course stressors shaping long-term HIV care. Poverty (34%) and non-specific “other” concerns (37%) predominated, followed by alcohol and substance abuse (2.7%), ART readiness challenges (2.6%), non-disclosure of HIV status (2.2%), risky sexual behaviours (2.1%), and denial (2.0%). Less common but notable was anxiety (1.1%), family dysfunction (1.5%), stigma (1.8%), and stress (1.5%), with bereavement, food insecurity, malnutrition, and spiritual concerns each affecting fewer than 1% of assessed individuals.

Findings indicate that while health-risk behaviours such as alcohol use and smoking remain relatively uncommon, a substantial proportion of PLWH experience poverty, psychosocial stressors, or mental-health vulnerabilities that may influence adherence and long-term treatment outcomes.

### Socio-economic characteristics

Socio-economic profiling revealed substantial structural vulnerabilities (Table 7). 53% of PLWH were married, predominantly in monogamous unions (36%), with smaller proportions separated (22%), single (13%), widowed (6.6%), or in polygamous marriages (5.1%). Minors accounted for 3.8% of the cohort.

**Table 7:** Socio-economic characteristics.

Variable	N	N = 54,348^1^
**Marital_Status_XX**	52,412	
Married		27,675 (53%)
Unknown		727 (1.4%)
Unmarried		24,010 (46%)
(NA)		1,936
**Marital_Status**	52,412	
Minor		2,017 (3.8%)
Cohabiting		3,714 (7.1%)
Separated		11,533 (22%)
Married		2,238 (4.3%)
Married_Mono		19,058 (36%)
Married_Poly		2,665 (5.1%)
Single		7,023 (13%)
Unknown		727 (1.4%)
Widowed		3,437 (6.6%)
(NA)		1,936
**Initial_Employment_XX**	49,295	
Regular_Income		8,124 (16%)
Irregular_Income		28,261 (57%)
Unemployed		10,329 (21%)
Unknown		2,581 (5.2%)
(NA)		5,053
**Initial_Employment**	49,295	
Boda_Boda		12 (<0.1%)
Casual_work		7,177 (15%)
Business		46 (<0.1%)
Minor		825 (1.7%)
Sex_Worker		2 (<0.1%)
Unemployed		9,504 (19%)
Employed		8,120 (16%)
Farmer		10 (<0.1%)
Mechanic		4 (<0.1%)
Unknown		2,581 (5.2%)
Peasant		13,338 (27%)
Vendor		7,587 (15%)
Vendor/Business Person		89 (0.2%)
(NA)		5,053
**Initial_Education**	48,245	
None		5,873 (12%)
Primary_or_Lower		27,403 (57%)
Secondary		11,871 (25%)
Higher_Institute		2,637 (5.5%)
Unknown		461 (1.0%)
(NA)		6,103
^1^n (%)		

Employment patterns revealed widespread income insecurity. At ART initiation, only 16% reported regular income, while 57% relied on irregular sources such as peasantry (27%), vending (15%), and casual work (15%); 21% were unemployed. By the most recent follow-up, 5.4% reported formal employment, reflecting low documented formal-sector within the care cohort.

Educational attainment was generally low: 57% had only primary schooling or less and 12% had no education at all. Secondary education was recorded in 25% and only 5.5% had attained higher institutional training.

### Programme status of cohort participants during the observation window

Programme status was available for all 54,348 clients. Overall, 29,695 (54.6%) were recorded as active in care, 2,375 (4.4%) as deceased, 12,192 (22.4%) as lost to follow-up (LTFU), 7,929 (14.6%) as transferred out and 2,157 (4.0%) had unknown status (Table 8). Status categories do not determine inclusion in descriptive summaries: baseline and follow-up variables remain available for clients who later transferred out, became LTFU, or died, and denominators therefore vary by variable and time point.

**Table 8:** Programme status of cohort participants during the observation period.

Variable	n(%) (N = 54,348)
Active	29,695 (54.6%)
Deceased	2,375 (4.4%)
LTFU	12,192 (22.4%)
Transferred	7,929 (14.6%)
Unknown	2,157 (4.0%)

Note: Categories reflect programme status as recorded in the TASO routine monitoring records during the observation window.

## Discussion

This decade-long analysis of 54,348 PLWH enrolled in TASO’s national programme provides one of the most comprehensive real-world portraits of HIV treatment and long-term care in Uganda. Spanning 11 sites across all major regions, the cohort captures how successive national and global ART policies; routine viral-load monitoring (2014), Universal Test and Treat (2017), and dolutegravir roll-out (2018); translated into population-level trends in ART initiation, immune recovery, regimen choice, and long-term outcomes.

The cohort was predominantly female (60%) with a median age near 40 years, and more than half aged 25–44 years, mirroring Uganda’s HIV epidemiology in which women of reproductive age remain disproportionately affected due to gender-specific vulnerabilities, including sociocultural and economic factors [12, 13]; and men typically present later for testing and treatment [14]. These patterns likely reflect a combination of gendered HIV risk, gender differences in care engagement, and the demographic maturity of TASO’s long-standing HIV care model. Age-by-policy analyses showed women consistently initiated antiretroviral therapy (ART) earlier and in greater numbers, underscoring gendered health-seeking patterns and continued testing gaps among men and adolescents [15]. Geographically, enrolment density reflected both historical burden and service availability, with urban and peri-urban sites such as Masaka, Mulago, Mbale, Jinja, Tororo, and Mbarara accounting for the largest share of PLWH [16, 17].

Uganda’s adoption of UTT was associated with earlier treatment and improved immune profiles. These findings are consistent with national and regional reports showing that “treat-all” policies reduce late presentation and mortality [18]. Body-mass-index trends indicated parallel nutritional recovery: underweight declined while overweight and obesity increased, signaling success in reversing HIV-associated wasting; a gradual shift toward non-communicable disease risk and the emerging metabolic risks documented in other long-term African cohorts, such as Tanzania [19]. However, given missingness, these findings should be interpreted as describing clients with recorded values rather than the full cohort.

Nonetheless, the persistence of severe immunosuppression among nearly one in five PLWH reveals ongoing barriers in HIV testing, linkage, and stigma that delay entry into care. Early identification of at-risk subgroups remains critical to align Uganda’s program with UNAIDS’ “95-95-95” goals [2].

Annual ART initiations peaked in 2014, coinciding with the introduction of routine VL monitoring and broader programme strengthening efforts (including expansion of testing and linkage to care); and again in 2018 when UTT and DTG roll-out converged, then declined steadily, consistent with a maturing, largely saturated treatment cohort. The most striking treatment trend was the shift from NNRTI-based to INSTI-based regimens after 2018, following the national rollout of DTG [20,21]. Although >99% of ART initiations in 2024 were INSTI-based (DTG-first-line policy), the 76% overall INSTI proportion reflects regimen class at the most recent recorded visit across the full cohort, including long-treated clients; overall, trends still indicate the rapid adoption of DTG regimens reported across sub-Saharan Africa [22]. Studies in Botswana and Nigeria reported similar trends, where DTG-based regimens rapidly became the standard of care [23].

With 97% of PLWH still on first-line therapy and very low use of second- or third-line regimens, the Ugandan programme demonstrates strong reliance and durability of first-line ART. The near-universal uptake of INSTIs strengthens the durability of Uganda’s ART program but also raises new priorities: monitoring weight gain, metabolic complications, and drug resistance dynamics, particularly as second-line PI-based regimens remain rare.

Classical opportunistic infections were infrequently recorded. Tuberculosis was the leading co-infection, but other opportunistic infections were each recorded in less than 5% of PLWH. In contrast, documented NCD comorbidity was present: hypertension (3.9%) and diabetes (2.5%) were the most frequently recorded, and overweight/obesity affected more than one-third of those with BMI data. Similar NCD emergence has been documented in other Ugandan and African HIV cohorts as ART coverage lengthens and PLWH age [24–26]. While TB remains salient, these findings support the need for integrated HIV–NCD services that address cardiovascular and metabolic risk alongside routine ART [23]. Nonetheless, the recorded prevalence of hypertension and diabetes may underestimate true burden because screening and documentation are not uniformly captured in routine records, including where measurements are not consistently available in TASO extractable fields.

Self-reported adherence was exceptionally high (≈97%), reflecting TASO’s long-standing investment in counselling and community support. Yet, as elsewhere, self-report likely overestimates true adherence [27,28]. Additionally, self-reporting is vulnerable to social-desirability bias and recall error compared with objective indicators such as pharmacy refill, pill counts, or viral-load suppression. Psychosocial assessments revealed substantial structural vulnerabilities: one-third of PLWH experienced poverty-related stressors, 8.7% had recorded depression, and smaller proportions reported stigma, denial, or risky sexual behaviour. Socio-economic data reinforce these risks, only 16% reported regular income at ART start and over half relied on informal or subsistence livelihoods, while education beyond primary school was uncommon. These indicators highlight that the majority of PLWH at TASO clinics are embedded in socio-economic contexts of poverty and informal livelihoods. Such conditions can limit healthcare access, undermine treatment adherence, and complicate long-term retention in care, [6,29]. These observations reinforce the need to integrate economic strengthening, mental-health care, and social protection into HIV service delivery. The high percentage of married individuals also reflects typical social structures in Uganda and suggests a potential impact of marital status on HIV transmission dynamics and ART adherence.

## Strengths and limitations

The key strengths of this study are its national scale, decade-long follow-up, and inclusion of demographic, clinical, behavioural, and socio-economic variables within a single routine dataset.

Limitations include incomplete data for some indicators (notably CD4 counts, BMI, and behavioural measures), reliance on self-reported adherence and risk behaviours, and potential misclassification within routine electronic records. Missingness was particularly high for CD4 and BMI: BMI could only be derived where both height and weight were recorded, and CD4 documentation varied across sites and periods as programme monitoring evolved. As missingness may not be random, estimates for these indicators may be biased and are interpreted cautiously.

Although TASO has wide geographic coverage, its sites are specialised centres of excellence and may differ from other ART delivery settings in Uganda; clients accessing TASO services may therefore not be fully representative of all people on ART nationally, and some estimates—particularly for comorbidity screening and service delivery indicators—may not generalise to lower-resourced facilities. While encouraging, adherence estimates warrant caution given reliance on self-report and routine programmatic categories, with potential social-desirability and recall bias—as well as survivorship/retention bias among PLWH remaining in care. The dataset did not include a consistently recorded measure of treatment interruption such as standardised pharmacy refill dates, visit interval fields, or interruption indicators across sites and years; we therefore did not quantify treatment gaps in this manuscript. Similarly, NCD/OI estimates should be interpreted cautiously given likely under-ascertainment in available records. Pregnancy and Prevention of Mother-to-Child Transmission (PMTCT) indicators were sparsely and inconsistently recorded in this extract and were therefore not analysed. Finally, as a retrospective secondary analysis of routine programme data, the study is descriptive and does not support causal inference.

## Policy implications and conclusion

Together, these findings illustrate how Uganda’s ART programme has matured: earlier initiation, widespread DTG adoption, improved clinical status, and high reported adherence. Yet persistent late presentation, entrenched poverty, mental-health burdens, and rising NCD risk delineate the next frontier for HIV care. Achieving and sustaining the UNAIDS 95–95–95 targets will require: (i) intensified HIV testing and early linkage, especially for men and adolescents; (ii) integrated HIV–NCD care with blood-pressure, diabetes, and nutritional screening embedded in ART clinics; and (iii) routine psychosocial and economic support to address poverty, depression, and stigma. Beyond Uganda, these findings reinforce calls for long-term, person-centred HIV programmes that combine potent ART with chronic-disease management to sustain epidemic control across sub-Saharan Africa.

By linking national policy innovations to population-level gains, and highlighting persistent structural and chronic-disease challenges, this study provides the empirical foundation for predictive modelling of virological failure and for designing person-centred, long-term HIV care in Uganda and across sub-Saharan Africa.

## Supporting information

S1 Data Supplementary variable dictionary. (XLSX)
